# Diagnostic challenges of arboviral infections and dengue virus serotype distribution in febrile patients in East Java, Indonesia

**DOI:** 10.1016/j.ijregi.2024.100512

**Published:** 2024-12-04

**Authors:** Do Duc Anh, Luthfiana Mutiara Sani, Rini Riyanti, Nurul Istinaroh, Truong Nhat My, Hoang Van Tong, Rike Oktarianti, Tran Thi Thanh Huyen, Le Huu Song, Kartika Senjarini, Thirumalaisamy P. Velavan

**Affiliations:** 1 Department of Biology, Faculty of Mathematics and Natural Sciences, University of Jember, Jember, Indonesia; 2 Institute of Tropical Medicine, University of Tübingen, Tübingen, Germany; 3 Vietnamese German Center for Medical Research (VG-CARE), Hanoi, Vietnam; 4 Faculty of Medicine, University of Jember, Jember, Indonesia; 5 108 Military Central Hospital, Hanoi, Vietnam; 6 Vietnam Military Medical University, Hanoi, Vietnam; 7 Faculty of Medicine, Duy Tan University, Da Nang, Vietnam

**Keywords:** Arboviruses, Dengue, Chikungunya, Zika, Serotypes, Indonesia

## Abstract

**Objectives:**

Arboviruses pose significant public health threats worldwide, with Southeast Asia being a hotspot for these infections. This study aimed to reassess the incidence of dengue, Zika, and chikungunya viruses in patients clinically diagnosed with dengue in East Java, Indonesia in 2023.

**Methods:**

The study included 108 patients admitted to hospitals in Jember, with blood samples collected on admission. Multiplex reverse transcription-polymerase chain reaction was used to detect viral RNA for dengue, Zika, and chikungunya, whereas dengue serotypes were identified using real-time polymerase chain reaction.

**Results:**

A total of 67 of 108 (62%) patients tested positive for dengue virus (DENV), one patient tested positive for chikungunya, and no cases of Zika were detected. Differences in laboratory parameters between patients who were DENV RNA–negative and confirmed dengue cases suggest possible misdiagnosis of dengue. Serotyping of DENV-positive samples revealed DENV serotype 3 as the predominant serotype in Jember, accounting for 34% of cases (n = 23 of 67), followed by DENV serotype 1 and DENV serotype 2 at 19% each (n = 13 of 67) and 6% for DENV-4 (n = 4 of 67), whereas 21% (n = 14 of 67) remained untyped.

**Conclusions:**

This study highlights the nature of the dengue outbreak in Jember in 2023, where all four DENV serotypes were in circulation, and underlines the need for serological or nucleic acid-based methods to improve arbovirus diagnosis in the region.

## Highlights

• Dengue dominated the 2023 hemorrhagic fever outbreak in Jember, Indonesia.
• Chikungunya was found in patients with dengue, but Zika was absent.
• Dengue virus serotype 3 emerging as the most common serotype.
• Clinical dengue diagnosis hints at misdiagnosis; serology or polymerase chain reaction is advised.

## Introduction

Vector-borne diseases account for over 17% of all infectious diseases worldwide, representing a significant public health threat [1]. In the Americas and Southeast Asia, the co-circulation of dengue, chikungunya, and Zika has emerged as an escalating epidemiological risk, marked by increasing case numbers, complications, and disease severity. These arboviral infections, transmitted primarily by *Aedes aegypti* and *Aedes albopictus*, can range from mild febrile illnesses to severe neurological complications. The overlapping transmission cycles of these viruses are especially common in Southeast Asia, where the warm, humid climate and high population density create optimal conditions for Aedes mosquitoes. This environment leads to frequent outbreaks, often peaking during the rainy season, which can place significant strain on already overburdened health care systems [2].

Dengue is hyperendemic in Southeast Asia, with cases increasing rapidly in the last decade [3]. Transmitted by the bites of *Aedes* mosquitoes, dengue is caused by one of four genetically distinct dengue virus (DENV) serotypes (DENV1-4). Severe forms of the disease, such as dengue hemorrhagic fever (DHF), were first documented in Indonesia during outbreaks in Jakarta and Surabaya in 1968 [4] and in Central Java in 1976, with the latter linked to DENV-3 [5]. In Indonesia, recurring dengue outbreaks, along with fluctuations in co-circulating DENV serotypes, continue to pose a major public health challenge and contribute to significant mortality [6,7]. Notably, DENV-4 emerged as the predominant serotype during the 2019-2020 outbreak in Jember, East Java [8].

Reports of Zika virus (ZIKV) circulation in Southeast Asia remain limited [9]. The first documented case of Zika in Indonesia dates to 1981 in Central Java [10], where patients were asymptomatic, complicating the diagnosis of febrile illnesses [11]. Sporadic reports of travelers returning from Indonesia with Zika infections further suggest underdiagnosis in the region [12,13]. The clinical overlap of Zika with other prevalent febrile illnesses, such as dengue and chikungunya, further complicates accurate diagnosis in Indonesia.

Chikungunya virus (CHIKV), of the *Alphavirus* family, is endemic to Africa and Asia. Transmitted by *Aedes* mosquitoes, it generally causes a mild, self-limiting illness. Indonesia experienced significant chikungunya outbreaks between 2009 and 2010, with 137,655 reported cases nationwide [14]. A considerable proportion of chikungunya, Zika, and dengue infections may present asymptomatically or with mild symptoms. When symptoms are present, they are often non-specific among the three etiologies: headache, myalgia, arthralgia, rash, and retro-orbital pain, making clinical differentiation difficult. Algorithms comparing clinical manifestations of these diseases have been proposed, but their sensitivity and specificity remain unvalidated [15].

Given this context, the current study re-evaluated febrile patients who presented with at least two clinical symptoms of viral hemorrhagic fever and were diagnosed as dengue cases by attending physicians. Using molecular diagnostics, the study reinvestigated the presence of dengue, Zika, and chikungunya and characterized the circulating pathogens by serotype.

## Materials and methods

### Study design and sample collection

A total of 108 febrile patients diagnosed with dengue, admitted to four hospitals in Jember, East Java Province, Indonesia, between January and July 2023, were included in this study. At the local hospitals, patients with dengue were diagnosed solely on the basis of clinical signs and symptoms*.* Inclusion criteria were febrile patients with a body temperature of ≥38°C for less than 7 days, accompanied by at least two clinical symptoms of dengue, such as headache, myalgia, retro-orbital pain, arthralgia/bone pain, or rash, characterized based on the World Health Organization South-East Asia Regional Office 2011 guidelines [16] (https://iris.who.int/handle/10665/204894). Blood samples were collected, and plasma was separated and stored at −20°C for further analysis. Conventional hematologic tests were carried out on admission.

### Ethical approval and consent to participate

Written informed consent was obtained from all hospitalized patients and/or their relatives, and from parents if subjects were under 18 years old. This study was approved by the research ethics committee at the University of Jember, Indonesia, with the reference number 1854/UN25.8/KEPK/DL/2023.

### RNA extraction and detection of dengue, Zika, and chikungunya viruses

Viral RNA was extracted from 140 µl of plasma using the QIAamp Viral RNA Mini Kit (Qiagen, Hilden, Germany) according to the manufacturer's instructions. The quality of the extracted RNA was assessed using a NanoDrop spectrophotometer (Thermo Fisher Scientific, Waltham, MA, USA). The RNA was then tested for the presence of DENV, ZIKV, and CHIKV RNA using the Fast Track Diagnostics (FTD) Zika/Dengue/Chikungunya Multiplex reverse transcription-polymerase chain reaction (RT-PCR) kit (Siemens Healthineers Company, Luxembourg). Briefly, the polymerase chain reaction (PCR) cycle conditions for FTD screening were as follows: reverse transcription at 50°C for 15 minutes, initial denaturation at 94°C for 1 minute, followed by 45 cycles of 8 seconds at 94°C and 1 minute at 60°C. Two master mixes were prepared: Master Mix 1 for ZIKV and Master Mix 2 for DENV and CHIKV. The reaction mix contained 1 µL of enzyme, 12.5 µl of buffer, and 1.5 µl of primer and probe. The mix was pipetted into a 96-well PCR plate, with 9 µl of sample and 1 µl of internal control. The plate was briefly mixed, and real-time PCR was performed using the LightCycler 480 Instrument II (Roche, Basel, Switzerland).

### DENV serotyping

DENV serotypes were identified using RealStar Dengue Type RT-PCR kit 1.0 (Altona Diagnostics, Hamburg, Germany) according to the manufacturer's instructions. Briefly, the PCR cycle conditions for DENV serotyping were as follows: reverse transcription at 55°C for 20 minutes, initial denaturation at 95°C for 2 minutes, followed by 45 cycles of 15 seconds at 95°C and 45 seconds at 55°C and 15 seconds at 72°C. Two master mixes were prepared: Master Mix 1 for DENV-1/4 and Master Mix 2 for DENV-2/3. The reaction mix contained 5 µl of Master A, 15 µl of Master B, and 1 µl of internal control 1. A total of 20 µl of the reaction mix was pipetted into a 96-well PCR plate, with 10 µl of sample. Reverse transcription and amplification cycles were conducted using the LightCycler 480 Instrument II (Roche, Basel, Switzerland).

### Statistical analysis

Categorical variables were reported as absolute counts and were compared using chi-square tests. Quantitative variables were presented as medians with ranges, and comparisons were made using Student's *t*-test or Mann–Whitney U tests as appropriate. The normality of distribution of the quantitative variables was tested using the Shapiro–Wilk test. All statistical analyses were conducted using R software version 4.3.2 (http://www.r-project.org). *P* <0.05 was considered statistically significant for statistical comparisons in the study.

## Results

### Detection of dengue, Zika, and chikungunya viruses

A total of 67 of 108 (62%) samples tested positive for DENV RNA, and one sample (1%) tested positive for CHIKV RNA using Multiplex RT-PCR. All tested samples were negative for ZIKV RNA. A total of 40 samples (n = 40; 37%) tested negative for DENV/ZIKV/CHIKV RNA (patients without DENV/ZIKV/CHIKV).

### Patient characteristics

Available data of the enrolled patients are summarized in Table 1. A total of 50 patients were male (46%) and 58 were female (54%), indicating an almost equal representation of both sexes in the study population. There was no significant difference in sex distribution between patients with confirmed infectious etiology (DENV) and patients without DENV/ZIKV/CHIKV. The clinical data and the age of the patients were not available for the analysis.

**Table 1:** Laboratory parameters of patients.

	Available patients (n = 70)	Non-DENV/ZIKV/CHIKV (n = 26)	DENV (n = 43)	CHIKV (n = 1)	*P*-value*
**Sex** (Male/Female)	40/30	14/12	25/18	Male	0.921
**RBC (**× 10^6^/µl)	4.67 [3.01, 6.24]	4.53 [3.01, 5.70]	4.78 [3.52, 6.24]	4.67	0.079
**HGB (**g/dl)	3.4 [8.7, 18.4]	13.0 [8.7, 15.8]	13.7 [9.20, 18.4]	13.1	0.065
**HCT (**%)	39.3 [25.6, 53.6]	37.8 [25.6, 46.5]	40.8 [26.5, 53.6]	37.8	**0.039**
**WBC (**× 10^6^/µl)	4.75 [1.2, 21.4]	5.70 [1.20, 21.4]	4.70 [1.30, 15.1]	5.2	0.437
**PLT (**× 10^3^/µl)	105.0 [13.0, 244.0]	119 [41.0, 244]	96.0 [13.0, 187]	119	0.160
**LYM (**%)	33.5 [3.0, 71.0]	29.0 [3.0, 56.0]	35.5 [8.0, 71.0]	32	0.388
**MONO (**%)	10.0 [2.0, 33.0]	11.0 [2.0, 29.0]	9.50 [6.0, 33.0]	7	0.960
**EO (**%)	0.0 [0.0, 14.0]	0 [0, 14.0]	0 [0, 9.0]	0	0.629
**BASO (**%)	1.0 [0.0, 4.0]	1.0 [0, 3.0]	1.0 [0, 4.0]	2	0.176
**NEUT (**%)	54.5 [10.0, 94.0]	57.0 [23.0, 94.0]	52.0 [10.0, 84.0]	59	0.512

BASO, basophil percentage; CHIKV, Chikungunya virus; DENV, Dengue virus; EO, eosinophil percentage; HCT, hematocrit; HGB, hemoglobin; LYM, lymphocyte percentage; MONO, monocyte percentage; NEUT, neutrophil percentage; RBC, red blood cell count; PLT, platelet count; WBC, white blood cell count; ZIKV, Zika virus.

Available data from 70 patients; variables are presented in median with range [min, max].

^a^*P*-value from the statistical comparison between the non-DENV/ZIKV/CHIKV group and the DENV group using the chi-square test, Student's *t*-test, or the Mann-Whitney U-test, as appropriate.

### Hematologic profile

The conventional blood laboratory tests from 70 of 108 patients were available for analysis. The results showed that the red blood cell count was within the normal range for the majority of patients (median [range] red blood cell = 4.67 [3.01-6.24]). However, the white blood cell (WBC) count was slightly decreased in overall, with the median WBC count close to the lower limit of the physiological range (median [range] WBC = 4.75 [1.2-21.4]), indicating possible viral infections.

In addition, some patients presented elevated levels of monocytes and eosinophils, suggesting chronic infection and/or inflammation (Table 1). Six patients (n = 6 of 70) had anemia, defined as hemoglobin levels below 12 g/dl, whereas a significant number (n = 62 of 70) had thrombocytopenia, with a platelet (PLT) count below 150,000 PLT/μl. The low median platelet count suggests the presence of DHF, with possible development of severe thrombocytopenia (Table 1).

Notably, comparing patients with confirmed DENV with patients without DENV/ZIKV/CHIKV, we found that hematocrit (HCT), an important marker of DHF, was significantly higher in patients with confirmed DENV (median = 40.8 [26.5-53.6]) than in the non-DENV/ZIKV/CHIKV group (median = 37.8 [25.6-46.5]) (*P* = 0.039). A higher median monocyte count was also observed in the non-DENV/ZIKV/CHIKV group than patients with confirmed DENV, although this difference was not statistically significant.

The patient with confirmed CHIKV infection had laboratory values within the normal range, except for a low platelet count (PLT = 119 × 10^3^/µl) indicating mild thrombocytopenia.

### Distribution of DENV serotypes

DENV-positive samples (n = 67 of 108) were subjected to serotype differentiation using the RealStar Dengue Type RT-PCR kit. The results indicated that n = 13 of 67 (19%) samples were identified as DENV-1, n = 13 of 67 (19%) samples as DENV-2, n = 23 of 67 (34%) samples as DENV-3, and n = 4 of 67 (6%) samples as DENV-4. In addition, n = 14 of 67 (21%) samples could not be assigned to any of the four DENV serotypes using RT-PCR methods (Table 2). No significant differences in the hematologic profile of patients were observed between the four DENV serotypes.

**Table 2:** Distribution of dengue, Zika, and chikungunya viruses among the patients.

Arbovirus detected	Febrile patients n =108 (%)
**Dengue (DENV)**	67 (62%)
DENV-1	13 (19%)
DENV-2	13 (19%)
DENV-3	23 (34%)
DENV-4	04 (06%)
Unidentified dengue serotypes	14 (21%)
**Chikungunya**	1 (1%)
**Zika**	0 (0)
Negative for dengue, Zika, and chikungunya	40 (37%)

DENV-1, 2, 3, 4: dengue virus serotype 1, 2, 3, and 4.

## Discussion

The results of this study provide insights into the ongoing co-circulation of dengue and chikungunya in Jember, Indonesia and highlight the complex landscape of arboviral infections in this hyperendemic region. Dengue remains a significant public health concern, with 62% of suspected cases testing positive and DENV-3 emerging as the predominant serotype during the 2023 outbreak. This is in line with previous studies that have shown fluctuations in serotype predominance in Indonesia over the years [6,7]. Although DENV-4 was predominant during the 2019-2020 outbreak in the region [8], our current study shows a shift toward DENV-3, emphasizing the dynamic nature of dengue transmission in Indonesia.

The absence of ZIKV detection is consistent with limited reports of Zika circulation in Southeast Asia [9]. However, the asymptomatic or mild presentation of Zika infections raises concerns about potential underdiagnosis, particularly, in regions such as Indonesia where clinical resources may be limited, and Zika symptoms overlap with other arboviral infections. The unspecific clinical symptoms of dengue, Zika, and chikungunya complicates accurate clinical diagnosis, further emphasizing the need for molecular diagnostics in such settings. This study identified a case of chikungunya infection, indicating the ongoing circulation of the virus. Previous research has also highlighted that chikungunya continues to be an underdiagnosed cause of acute febrile illness in Indonesia [17].

The hematologic findings in this study align with known markers of dengue infections. Most notably, the significantly higher HCT levels observed in patients with confirmed DENV compared with non-DENV/ZIKV/CHIKV cases support the utility of HCT as a diagnostic marker for DHF. In addition, the widespread occurrence of thrombocytopenia in DENV patients is consistent with typical clinical presentations of dengue and is a well-established marker for identifying potential severe cases. The high prevalence of thrombocytopenia also suggests that some patients may have progressed to severe dengue, warranting close clinical monitoring. However, the clinical data of the patients were not available for the analysis. To confirm DENV infections and prevent misdiagnosis, laboratory diagnostics are essential [18], as demonstrated by the significantly lower HCT levels in patients without detectable DENV/ZIKV/CHIKV RNA than in patients with confirmed DENV. Rapid diagnostic tests (for non-structural protein 1, immunoglobulin G, and immunoglobulin M) can reduce false positives despite cross-reactivity within the Flaviviridae family [19] and aid in classifying primary and secondary dengue infections [20], which can support disease management. Furthermore, nucleic acid testing for DENV detection and serotype identification offer high specificity and sensitivity, making these methods valuable, particularly, during the early phase of infection [21].

Globalization has been linked to the rapid spread of diseases, whereas climate change has contributed to the geographic expansion of arthropod-borne vectors [22]. Reports have highlighted ZIKV, yellow fever virus, Japanese encephalitis virus, West Nile virus, tick-borne encephalitis virus, and DENV as predominant arboviral infections. Indonesia, a hyperendemic country for dengue, has seen a rising number of cases over the past decade [23], with 143,266 cases reported in 2022 across 467 cities and regions, resulting in an incidence rate of 42.25 per 100,000 population. Although research on DENV is well-documented in Surabaya, one of Indonesia's largest cities, there is a lack of studies in other areas, including Jember Regency, the third largest city in East Java. Reports show that Jember experienced the highest number of cases in 2019 (988 cases), with a significant decline in 2021 (447 cases), followed by an increase to 781 cases in 2022 [8]. The highest cases in Jember were reported in Kaliwates, where our study samples were collected.

The factors influencing dengue severity include host and viral characteristics, particularly, the infecting DENV serotype [24]. In Indonesia, DENV-3 caused dengue epidemics since its identification in 1970 until it was displaced by DENV-1 and DENV-2 in the early 2000s [23]. Although all DENV serotypes are endemic in Indonesia, DENV-3 has been reported to cause severe infections, including encephalitis [25]. In addition, the predominance of DENV serotypes varies across the Southeast Asia from 2021 to 2023, with Vietnam reporting DENV-2 [26], Singapore reporting DENV-3 [27], and Malaysia reporting DENV-4 [28]. This diversity raises concerns because shifts in predominant serotypes could impact the intensity of future outbreaks due to the phenomenon of antibody-dependent enhancement [29].

One of the key limitations of this study is the inability to assign 21% of DENV-positive samples to any of the four serotypes, which may be attributed to low viral loads in these samples. Further investigation using next-generation sequencing or alternative molecular techniques may be necessary to better understand the genetic diversity of circulating DENV serotypes. Furthermore, our study focused only on symptomatic patients presenting at local hospitals, which limits the generalizability of the findings. Therefore, further studies on dengue seroprevalence and vector surveillance could provide a broader and more comprehensive epidemiological overview of dengue and other arboviruses.

## Conclusion

In conclusion, this study emphasizes the need for ongoing surveillance and molecular diagnostics to accurately detect and differentiate between arboviral infections in hyperendemic regions such as Indonesia. The shift in DENV serotype predominance, together with the potential for co-infections and misdiagnosis, underscores the need to improve diagnostic capabilities. This improvement is not only important to optimize patient outcomes but also to effectively support public health.

## Funding

This study was funded by the PAN-ASEAN Coalition for Epidemic and Outbreak Preparedness (German Academic Exchange Service Project ID: 57592343). The funder has no role in the study design, data collection and analysis, decision to publish, or preparation of the manuscript.

## Ethical approval

Written informed consent was obtained from all hospitalized patients and/or their relatives and from parents if subjects were under 18 years old. This study was approved by the research ethics committee at the University of Jember, Indonesia, with the reference number 1854/UN25.8/KEPK/DL/2023.

## Author contributions

TPV and KS conceptualized and designed the study. TPV contributed to the study materials and assays. RR recruited the patients. LMS, DDA, NI, and TTTH performed the experimental procedures. DDA and HVT were involved in the statistical analysis and validation of the results. TNM, TPV, RO, TTTH, and LHS supervised experimental procedures. TPV, HVT, and DDA wrote the first draft. All authors have read and approved the manuscript.

## Declarations of competing interest

The authors have no competing interests to declare.
